# The contribution of posterior circulation to memory function during the intracarotid amobarbital procedure

**DOI:** 10.1007/s00415-011-6391-4

**Published:** 2012-01-26

**Authors:** M. Zijlmans, C. J. A. Huibers, G. J. Huiskamp, G. A. P. de Kort, W. C. J. Alpherts, F. S. S. Leijten, J. Hendrikse

**Affiliations:** 1 Department of Neurology and Clinical Neurophysiology, Rudolf Magnus Institute of Neuroscience, University Medical Center Utrecht, Utrecht, The Netherlands; 2 Department of Radiology, University Medical Center Utrecht, Utrecht, The Netherlands; 3 Department of Psychology, The Epilepsy Institutes of the Netherlands Foundation (SEIN), Heemstede, The Netherlands

**Keywords:** Wada test, Amobarbital, Foetal type, Willis, Cerebral arteries

## Abstract

The purpose of this study was to evaluate the contribution of posterior circulation to memory function by comparing memory scores between patients with and without a foetal-type posterior cerebral artery (FTP) during the intracarotid amobarbital procedure (IAP) in epilepsy patients. Patients undergoing bilateral IAP between January 2004 and January 2010 were retrospectively included. Pre-test angiograms were assessed for the presence of a FTP. Memory function scores (% correct) after right and left injections were obtained. Functional significance of FTP was affirmed by relative occipital versus parietal EEG slow-wave increase during IAP. Memory and EEG scores were compared between patients with and without FTP (Mann–Whitney *U* test). A total of 106 patients were included, 73 with posterior cerebral arteries (PCA) without FTP (‘non-FTP’), 28 patients with unilateral FTP and 5 with a bilateral FTP. Memory scores were lower when amytal was injected to the hemisphere contralateral to the presumed seizure focus (on the right decreasing from 98.3 to 59.1, and on the left decreasing from 89.1 to 72.4; *p* < 0.001). When IAP was performed on the side of FTP memory scores were significantly lower (70.8) compared to non-FTP (82.0; *p* = 0.02). Relative occipital EEG changes were 0.44 for FTP cases and 0.36 for non-FTP patients (*p* = 0.01). A relationship between vasculature and brain function was demonstrated by lower memory scores and more slow-wave activity on occipital EEG during IAP in patients with foetal-type PCA compared to patients with non-FTP. This suggests an important contribution of brain areas supplied by the PCA to memory function.

## Introduction

The intracarotid amobarbital procedure (IAP), or Wada test, is used in patients with intractable temporal lobe epilepsy to determine language lateralization and assess the risk of postoperative global amnesia. Amobarbital temporarily inactivates brain tissue distal to the injection site [1, 2]. The Wada test demands conscious recollection of verbal and visuospatial information from long-term memory. During this test the effect of amobarbital is evaluated with EEG.

The hippocampus is vascularised by branches from the middle and posterior cerebral arteries (MCA and PCA). People with trombo-embolic disease of the PCA often show memory deficits [3]. However, the precise contribution of the posterior part of the hippocampus to memory function is unclear. Considerable variability exists in the vascularisation of the brain. In the complete configuration of the circle of Willis, the anterior cerebral artery (ACA) and MCA are supplied by the internal carotid artery (ICA) and the PCA by the vertebrobasilar system. In foetal-type PCA (FTP), the embryonic derivation of the PCA from the ICA has persisted. Consequently, the entire ipsilateral hemisphere depends on blood supply from the ICA. Unilateral FTP is present in about 25% of cases, the bilateral variant in 7–10% [4, 5].

**Fig. 1:**
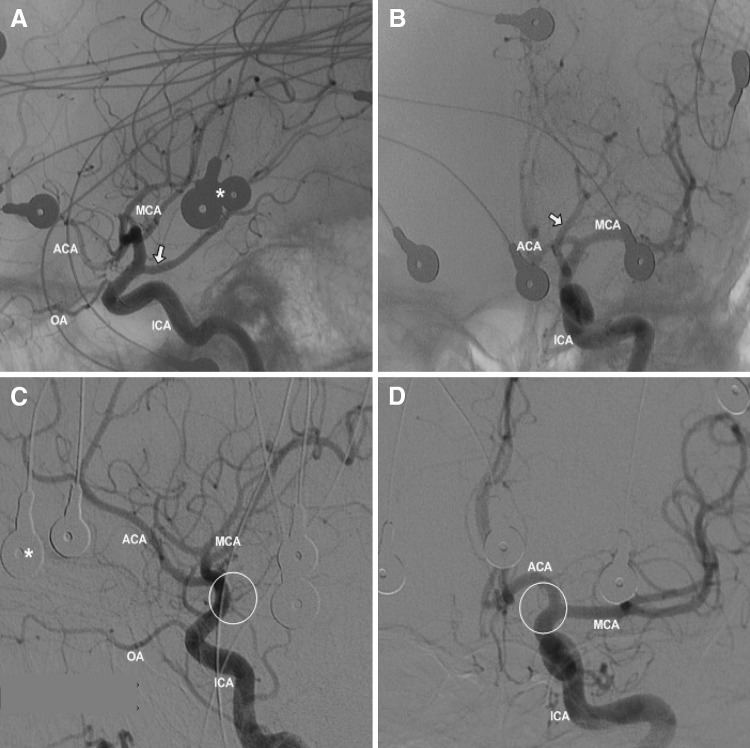
Intracarotid angiography. Left intracarotid angiography in foetal-type PCA (FTP) (**a**, **b**) and no-FTP (**c**, **d**) patients. **a** Sagittal and **b** coronal: PCA (*white arrow*) filling via the posterior communicating artery from the ICA resulting in complete filling of the posterior circulation, indicating the presence of an FTP. **c** Sagittal and **d** coronal: no contrast in the posterior circulation (*white circle*), indicating a PCA without FTP. *ICA* internal carotid artery, *ACA* anterior cerebral artery, *MCA* middle cerebral artery, *OA* ophthalmic artery, *asterisk* EEG electrode

Consequently, amobarbital affects the complete ipsilateral hemisphere in patients with FTP, whereas in non-FTP only the ACA and MCA supplied areas are involved. This might yield differences in memory function, because of the double vascularisation of the hippocampus. The aim of the present study is to evaluate the contribution of the PCA supplied brain areas to memory function by comparing memory scores during the IAP between patients with and without FTP (Fig. 1).

## Methods

### Patients

Our retrospective analysis included patients with intractable (temporal lobe) epilepsy who were evaluated as potential surgical candidates and underwent the IAP to determine memory and language lateralization. Patient data were retrieved from the Dutch Collaborative Epilepsy Surgery Program database and the electronic hospital information system. The study was approved by the Medical Ethical Committee of the University Medical Centre Utrecht.

This study included patients in the period between January 2004 and January 2010 who met the following criteria: (a) bilateral IAP performed at the University Medical Centre Utrecht, (b) angiograms available in the Picture Archiving and Communication System, and (c) EEG recordings digitally retrievable. No exclusion criteria were stated in advance.

### Intracarotid amobarbital procedure

All patients underwent bilateral intracarotid angiography as a routine component of the IAP by transfemoral catheterization of the internal carotid artery, in order to visualize the cerebral vascularisation pattern and thereby the correct position of the catheter before injecting the amobarbital by hand. The performing radiologist reported the presence or absence of a foetal-type PCA.

The presumed seizure focus side was injected first, in general 125 mg amobarbital was administered; in children this was occasionally 100 mg, depending on age. Language function was assessed during the first 2–3 min. Memory function (recollection) was assessed by presenting five items (three visual and two auditory) during the amobarbital effect, usually 2.5 min after injection, and recollecting these items 15 min after injection. Memory score was graded as a percentage. A cut-off score of 50% was used for passing or failing the test. After about 30 min the procedure was repeated on the contralateral side and language dominance was stated as either left or right, or bilateral [6].

### Angiography

All angiograms were independently evaluated by two neuroradiologists for the presence or absence of an FTP and cross-filling. In cases of disagreement, the angiograms were reviewed by both in order to reach consensus.

### Electroencephalography

We analyzed occipital EEG changes to confirm the hypothesis that foetal-type PCA results in altered brain function in the posterior brain areas during amobarbital injection.

During the IAP, brain activity was continuously monitored using a 22-channel EEG from approximately 30 min before until 15 min after the procedure (Micromed^®^, Treviso, Italy). These EEG data were retrieved from the database and visually inspected for this study. Three different parts of the EEG were analyzed in every patient. From baseline EEG and EEG after right and left amobarbital injections, approximately 60 s of artefact-free EEG (i.e. without muscle artefacts or baseline shifts) were selected for analysis. If 60 s of uninterrupted artefact-free EEG were not available, two parts of 30 s were selected. Any remaining artefacts were removed stepwise from the analysis.

We hypothesized that patients with an FTP would have considerably more EEG slowing over the ipsilateral occipital areas after amobarbital injection, compared to patients with a non-FTP vascularisation pattern. In the non-FTP configuration of the circle of Willis, the amobarbital would not reach the posterior circulation in substantial amounts. Therefore, occipital EEG effects would be minimal in these subjects.

Parietal electrodes P3 and P4 were selected to reflect amobarbital effects in the vascularisation area of the MCA; occipital electrodes O1 and O2 were selected for PCA areas. Frontal electrodes were not chosen because of potential eye movement artefacts and temporal electrodes were avoided because of muscle artefacts. We chose the contralateral parietal electrode as a reference (i.e. P3 for right-sided injections and P4 for left-sided injections).

A frequency decomposition (Fourier analysis) of the EEG data was made to quantify slow brain activity. Slow activity was defined as 1–7 Hz. We chose to include both delta and theta activity as both these bands are normally absent in awake adults. In young children however, and in several types of brain pathology, persistent cortical theta wave activity can be observed. In order to correct for these non-amobarbital related theta waves, the baseline EEG was incorporated in the calculation.

A low pass filter was set at 70 Hz, while the high pass filter was 0.53 Hz. Absolute power was calculated in 5 s epochs for 1–7 Hz. This was carried out in all patients for the 60 s (12 epochs) baseline EEG and 60 s after left and right amobarbital injections. Mean absolute power baseline values were subtracted from mean values after injection. This resulted in an absolute power difference after amobarbital injection.

To standardize the effect for group comparison, the absolute power change in the occipital area was divided by the absolute power change in the parietal area on the same side, ipsilateral to injection. We expected that a relative power change would range between zero and one, taking into account that the absolute power change in the parietal region is likely to be greater than that in the occipital region in most patients. A relative power change of one indicates that the magnitude of amytal-induced EEG slowing in the occipital region was equal to that in the parietal region. A relative power change of zero indicates that the magnitude of EEG slowing in the occipital region was minimal.

### Statistical analysis

Continuous data were expressed as mean ± SD. Data were analysed using SPSS software version 15.0 for Windows (SPSS Inc., Chicago, IL). Baseline variables such as age, gender, underlying diagnosis, first injection side, amobarbital dose and language lateralization were compared for patients with FTP and patients with non-FTP by χ^2^ and Fisher’s exact test for categorical variables and Mann–Whitney *U* test and two-sided independent sample *t* tests for continuous variables.

Analysis was first by hemisphere and then by presence of FTP on either side. In the first case, patients with right or left FTP were compared with those having no FTP on that same side. In the overall analysis all FTPs (left, right or bilateral) were compared with all non-FTP posterior circulations. The presence or absence of FTP was correlated with occipital EEG effect scores (0.0–1.0), reaction time (in minutes) and memory scores (in % recall). We also investigated whether it made a difference if FTP was on the side of the epileptic focus or on the healthy side. The statistical test was chosen based on the skewness of the probability distribution. If data were normally distributed, a *t* test (two-sided) was chosen; otherwise the Mann–Whitney *U* test was used. Reaction time was also divided into categories (0–3 min and >3 min) and compared using Fisher’s exact test. A *p* value <0.05 was considered statistically significant.

## Results

### Patients

A total of 106 patients (57 male and 49 female subjects) were included. Mean age at IAP was 32.8 ± 12.2 years (range 10–63 years). The primary underlying diagnosis was mesial temporal sclerosis (MTS) in 47 (43%) patients, brain tumours (one malignant) in 31 (29%) patients, (cortical) dysplasia in 14 (13.2%) patients and 2 patients had epilepsy due to trauma. Twelve (11%) patients had a different or unclear underlying pathology. Seventy-three (68.8%) patients had a non-FTP posterior circulation (i.e. PCA arising from the vertebrobasilar system). Eleven (10.4%) patients had left foetal-type posterior circulation, 17 (16%) patients had right FTP and 5 (4.7%) patients had bilateral FTPs. Baseline characteristics including gender, age, primary diagnosis, side of first injection and language lateralization were similarly distributed between both groups (Table 1).

**Table 1:** Patient characteristics for non-FTP and FTP patients at baseline

Baseline characteristics	Non-foetal type PCA (*n* = 73)	Foetal type PCA (*n* = 33)	*p* value
Gender			
Male	33 (44.2)	16 (48.5)	0.83
Female	40 (54.8)	17 (51.5)	
Age, years (range)	33.2 ± 12.2 (10–63)	32.0 ± 12.2 (14–58)	0.65
Epileptic focus			
Temporal	69 (94.5)	32 (97.0)	0.58
Extratemporal	4 (5.5)	1 (3.0)	
Primary diagnosis			
MTS	35 (47.9)	12 (36.4)	0.37
Low-grade glioma	16 (21.9)	8 (24.2)	
High-grade glioma	1 (1.4)	0	
Hemangioma	4 (5.5)	2 (6.1)	
Dysplasia	10 (13.7)	4 (12.4)	
Trauma	0	2 (6.1)	
Other or unknown	7 (9.6)	5 (15.1)	
Seizure focus side			
Left	30 (44.1)	16 (50.0)	0.67
Right	38 (55.9)	16 (50.0)	
Amobarbital dose (mg)	120.2 ± 25.9	122.3 ± 12.0	0.64
Language lateralization			
Left	64 (90.1)	30 (93.8)	0.75
Right	3 (4.2)	2 (6.3)	
Bilateral	4 (5.6)	0 (0.0)	
Posterior circulation			
No FTP	73 (68.9)		
Left foetal type PCA		11 (10.4)	n/a
Right foetal type PCA		17 (16)	
Bilateral foetal type PCA		5 (4.7)	

Data are expressed as number of patients with variable present, *n* (%) and mean ± SD

*n*/*a* not applicable

### Memory score

When the seizure focus side was injected, memory scores were significantly higher compared to memory scores after contralateral injection. In patients with right-sided foci scores were 98.3 after right injection and 59.1 after contralateral injection (*p* < 0.001.) For left-sided foci scores were 89.1 versus 72.4 (*p* < 0.001). Memory scores were lower in FTP cases, whether this was on the seizure focus side or not (Table 2). After right injection, the memory score in the FTP was 77.7, and 88.9 in non-FTP cases (*p* = 0.06). After left injection, the score was 61.3 in the FTP and 75.4 in the non-FTP group (*p* = 0.05). In overall analysis scores were 70.8 and 82.0, respectively (*p* = 0.02, Table 2).

**Table 2:** Results after left and right amobarbital injections and grouped results for non-FTP and FTP cases

	Left			Right			Bilateral		
	No FTP	FTP	*p* value	No FTP	FTP	*p* value	No FTP	FTP	*p* value
Seizure focus									
Left	47 (55.3)	7 (46.7)	0.58	41 (52.6)	13 (59.1)	0.64			n/a
Right	38 (44.7)	8 (53.3)		37 (47.4)	9 (40.9)				
Reaction time (min)	3.37 ± 0.78	3.60 ± 0.91	0.33	3.13 ± 0.68	3.19 ± 0.40	0.23	3.26 ± 0.73	3.38 ± 0.68	0.22
Reaction time									
0–3 min	59 (69.4)	9 (60.0)	0.55	68 (87.2)	17 (77.3)	0.31	127 (77.9)	26 (70.3)	0.39
>3 min	26 (30.6)	6 (40.0)		10 (12.8)	5 (22.3)		36 (22.1)	11 (29.7)	
Memory score									
Total	75.4 ± 28.6	61.3 ± 29.6	0.05*	88.9 ± 21.2	77.7 ± 29.3	0.06	82.0 ± 26.1	70.8 ± 30.2	0.02*
Focus side injection	91.1 ± 13.7	80.0 ± 21.4	0.11	99.0 ± 4.4	96.2 ± 7.7	0.06			
Contralateral injection	61.1 ± 31.3	45.7 ± 25.1	0.18	77.6 ± 24.4	51.1 ± 28.5	0.02*			
EEG score	0.35 ± 0.14	0.47 ± 0.20	0.02*	0.35 ± 0.14	0.43 ± 0.17	0.08	0.36 ± 0.15	0.44 ± 0.19	0.01*

Data are expressed as number of patients with variable present, *n* (%) or mean ± SD

*n*/*a* not applicable

* *p* < 0.05

### EEG score

For all patients, both left and right occipital EEG effect scores were calculated. A higher score indicates a greater increase in slow wave activity over the ipsilateral occipital lobe. In 7 patients EEG scores of only one side could be calculated, which were included in the analysis. Five outliers with EEG scores exceeding the theoretical maximum of 1.0 (range 1.1–2.2) were excluded.

EEG scores for left-sided injections were 0.47 with FTP and 0.35 without FTP (*p* = 0.02). For right-sided injections these scores were 0.43 and 0.35, respectively (*p* = 0.08). Taking right and left together, scores of 0.44 and 0.36 were found (*p* = 0.01).

## Discussion

This study shows that the presence of foetal-type posterior circulation is related to lower recognition memory scores during the IAP compared to no foetal-type posterior circulation. EEG supports that FTP results in altered posterior brain function after amobarbital injection compared to non-FTP vascularisation.

This strongly suggests a role of posterior circulation supplying brain areas involved in memory function, as previously suggested [7]. The anterior choroidal artery, generally originating from the ICA, is an important branch supplying the medial temporal region, including the hippocampus. However, the occipital two-thirds of the hippocampus is supplied by PCA branches [8]. In case of no FTP, the areas supplied by the PCA will remain functionally active during intracarotid amobarbital injection, whereas in case of an FTP the entire hemisphere will be affected, securing total inactivation of ipsilateral memory function. Therefore, theoretically, in FTP cases more reliable memory scores would be achieved as this score will reflect pure contralateral memory function. This could theoretically provide a better prediction of post-operative memory loss. One could argue whether these memory scores are solid enough in patients without an FTP, as part of this score may be obtained by ipsilateral memory function in the posterior. Whether postoperative amnesia is worse in these patients has yet to be determined in future research.

Although the hippocampus plays a key role in memory function, other possible explanations for lower memory scores in FTP cases should be taken into consideration. For instance, other brain areas supplied by the PCA, not directly related to memory function, could influence IAP results in various ways. The PCA often supplies the upper brain stem affecting eye movement, the primary visual cortex and the posterior thalamus where visual connections reside. Amobarbital flow to these areas would compromise the patient’s ability to visually attend to objects being presented and could therefore result in lower memory scores. In addition, the effect could be an impaired language function instead of memory function in the dominant hemisphere. However, during WADA tests, the examining neuropsychologists are aware of possible visual problems and avoid the potentially impaired side when offering their tests; they also test for language. Therefore, an impaired function of the posterior hippocampus supplied by the PCA seems a likely explanation.

That presence of an FTP has a functional meaning is proven by the higher occipital EEG effect score in FTP cases, corresponding to a larger increase in slow wave activity over the ipsilateral occipital area after injection. This suggests that the amobarbital effect extends to the posterior cortex supplied by the FTP. However, this may not be the only factor, as in cases of no FTP occipital EEG alterations are also found, although to a lesser extent. This may be due to volume conduction or indirect effects, e.g. through the thalamus which sustains connections with the occipital lobe.

Over the past few years the IAP has been criticized for its reliability and validity to determine memory lateralization, especially when the language-dominant hemisphere is injected [9]. This is further supported by questions about the test–retest reliability [10]. Two studies have reported false-positive (patients failed the IAP but did not suffer from postoperative amnesia) and false-negative (patients passed the IAP but presented post-operative amnesia) IAP results [11, 12]. To the best of our knowledge, memory function during IAP has never been assessed in relation to the presence or absence of an FTP. In a recent study the presence or absence of an FTP was related to transient shivering during IAP and thermoregulation of the thalamus [13]. Memory function was not addressed in this previous study.

A few studies have reported on expected and observed amobarbital distribution, e.g. by simultaneous radioactive 99mTc-HMPAO injection during the IAP [14, 15]. In cases where patients passed the IAP test, while the hippocampus was not inactivated according to SPECT, 13% developed postoperative memory deficits [15]. The hippocampus was found functional in 60% of cases. This might be explained by the variation in vascularisation patterns we found. Because the hippocampus plays a key role in memory function, incomplete hippocampal inactivation during IAP in patients without FTP could account for the higher memory scores in this group compared with FTP cases.

Some authors have refuted the idea that the PCA contribution to hippocampal vascularisation poses a major problem for the IAP, and is a cause of unreliable memory scores. In a previous study, functional effects in the ipsilateral posterior hippocampus were found on both SPECT and intracranial EEG recordings during IAP, while only the anterior hippocampus was perfused with amobarbital [16]. Thus, angiography does not provide a reliable estimation of the amobarbital effect. They studied contralateral EEG effects by amobarbital injection, and concluded that this was irrespective of the presence or absence of anterior cerebral artery cross-filling on angiography. This suggests that amobarbital may exert functional effects distant from where it is distributed, in this case due to a functional disconnection or loss of neuronal commissural input, resulting in reduction in regional cerebral blood flow and the appearance of slow waves on the EEG in the non-injected frontal lobe. These results were confirmed in another study [1]. The occurrence of contralateral EEG effects was not related to cross-perfusion of amobarbital and appeared to be a transient functional disconnection from one hemisphere to another. Gotman et al. [17] quantified delta activity by intracerebral EEGs during the IAP. There was an increase in delta activity in all ipsilateral areas, including the occipital lobes, and to a lesser extent in contralateral structures. The ipsilateral hippocampus had delta waves in over 90% of injections. They argue that the slow waves may not be caused by a direct effect of the drug, but rather by a functional deafferentation due to the profound inactivation of structures surrounding the hippocampus.

Urbach et al. [18] observed perfusion of the entire hippocampus only in hemispheres with a foetal origin of the PCA. Hippocampal activity measured by depth electrodes changed under the influence of amobarbital, but did not differ between anterior and posterior parts, while SPECT showed perfusion of the anterior hippocampus only. Also in this previous study, the authors argue that EEG effects on the posterior hippocampus during IAP can occur without direct perfusion of those brain areas. However, our findings indicate that in patients with a foetal-type PCA occipital slow wave activity is more distinct and, most importantly, memory function scores are lower compared to patients without PCA filling. This is a more direct proof than can be provided to the contrary by the above anatomical studies.

A limitation of our study may be that patients with posterior communicating artery filling but minimal PCA filling on angiography have been included in the non-FTP group. Although these patients did not have an FTP, amobarbital could still have reached the occipital areas. Carotid contributions to the PCA in these non-FTP cases would have resulted in an underestimation of the effect of FTP on memory and thus have strengthened the conclusions of the current study.

Our results of lower memory function scores in the presence of an FTP have clinical consequences for epilepsy patients undergoing the IAP, and their physicians. In the majority of patients who do not have an FTP, memory scores should be interpreted with caution, as they may partly reflect remaining ipsilateral hippocampal function. Other memory lateralization tests besides the IAP should be considered to rule out memory localization ipsilateral to the seizure focus side to prevent postoperative amnesia.
